# A Cross-Sectional Analysis of Plastic Surgery With GLP-1-Related Content on TikTok

**DOI:** 10.1093/asjof/ojag094

**Published:** 2026-05-20

**Authors:** Rohun Gupta, Sonia S Patel, Atharva M Bhagwat, Rushil Gupta, Thomas Y Xia, Herluf Lund, Sumesh Kaswan

## Abstract

TikTok has risen to the forefront of social media platforms, becoming an increasingly popular source for health information dissemination. The utilization of glucagon-like-peptide-1 (GLP-1) agonists has resulted in a considerable increase in cosmetic plastic surgery interest. This study aims to analyze TikTok content related to medical weight loss medications and plastic surgery. TikTok was queried using several phrases to assess plastic surgery and weight loss related in November 2025. Video statistics and characteristics were reviewed and graded using the validated DISCERN tool for the top 50 videos from each search algorithm. *P*-values <.05 were noted to be statistically significant. A total of 101 videos met inclusion criteria with a total of 12,662,385 views, 318,749 likes, 1033 comments, and 54,245 shares. Most videos were produced by physicians (57.4%) followed by patients (23.8%). When stratified by content creator type, median DISCERN scores were similar across groups, although nonphysician healthcare providers had the numerically highest median score (*P* = .280; ε^2^ = 0.011). Educational videos had statistically significantly greater DISCERN scores for items assessing videos’ description of how treatments work, benefits of treatments, and outcomes if no treatment was used. This study found that social media platforms can help disseminate healthcare information. Although content varies across creator and video category subtypes, overall quality has improved and creators should look to provide qualified sources of healthcare information, foster an environment of shared decision-making, and for providers to discuss how GLP-1 medications and plastic surgery may affect quality of life.

The launch of TikTok (Culver City, CA) in 2016 marked a pivotal shift in social media content and engagement. The application's unique short-form, algorithm-driven video content quickly attracted a large audience, with over 1.12 billion monthly users worldwide with over 150 million monthly users in the USA alone.^1,2^ TikTok is one of the most influential social media platforms with a wide variety of content creators and subject matters, ranging from comedic and trending videos to videos related to health information. Several prior studies have demonstrated that the platform is increasingly being utilized as a resource for dissemination of medical information.^3^ A study by Kirkpatrick and Lawrie found that the majority of individuals who had utilized TikTok have obtained health information both unintentionally and intentionally.^4^ TikTok has several advantages for health-related material including ease and accessibility to information, widespread reach/access to information, and personalized information from both healthcare providers and patients. Still, there are several disadvantages that need to be taken into account including the spread of misinformation, lack of regulation protocols, and commercial interests that may be paid promotions rather than factual medical information.

Medical management has been a highly popular method for weight loss, with amphetamine type stimulants being introduced in the early 1940s.^5^ Since then, there have been countless medications that have been introduced that have been effective in weight loss. Ozempic (Novo Nordisk, Bagsværd, Denmark), perhaps one of the most popular weight loss medications in recent years, is a glucagon-like-peptide-1 (GLP-1) agonist that was approved by the Food and Drug Administration (FDA) in 2017 for the management of Type 2 Diabetes, was also noted to be highly effective in weight loss.^6^ The active ingredient in ozempic, semaglutide, was then approved by the FDA in 2021 for weight loss in individuals with obesity.^7-9^

The success in achieving significant reductions in weight, waist circumference, and waist-to-height secondary to weight loss medications has resulted in increased interest in management of the soft tissue sequelae including increased interest in plastic surgery procedures.^10^ Additionally, with the increasing rate of obesity in the USA, it is highly likely that patients will turn to medical management of weight loss, possibly increasing the demand for aesthetic based procedures.^11^ Furthermore, several studies have found that adults have utilized social media sources as a means to obtain weight loss information.^12^

Given the popularity of social media platforms such as TikTok and the uptrend in medical management of weight loss and plastic surgery-related procedures, this study aims to analyze TikTok content related to plastic surgery and GLP-1 agonist type medications to improve patient education. To date, there have been no published studies that have looked to elucidate the medical information related to GLP-1 agonists and plastic surgery.

## METHODS

TikTok was searched in November of 2025 using several phrases including “Ozempic plastic surgery,” “Wegovy plastic surgery,” “semaglutide plastic surgery,” and “GLP-1 plastic surgery.” The search terms were derived from the combination of popular weight loss medications and plastic surgery in order to garner adequate video retrieval. A total of 4 search terms were selected arbitrarily in order to attempt to include as many top videos regarding the topic. The top 50 videos from each search query were then analyzed. Exclusion criteria included videos that did not pertain to weight loss medications or plastic surgery or if the videos were commentaries, duplicates, or nonrelevant. Video statistics and characteristics were reviewed and graded by 2 independent reviewers using DISCERN, a validated 16-question scoring instrument used to assess the quality of consumer health information.^13^ Section one of DISCERN evaluates reliability, while section 2 assesses the quality of information on treatment choices, and section three provides an overall rating of the publication. Statistical analyses were performed in GraphPad Prism 10.6.0 (GraphPad LLC, Boston, MA). The Shapiro–Wilk test revealed that while some data were normally distributed, others were not; consequently, analyses were conducted without assumptions of normality. One-way ANOVA was conducted with Kruskal–Wallis test followed by Dunn's multiple comparison test. Groups with only one data point were excluded from statistical analyses. Significance was set at α = 0.05.

## RESULTS

A total of 200 videos were reviewed, with 91 not meeting inclusion criteria, and 8 being excluded due to duplicates, bringing the total number of videos included in this study to 101. There were a combined 12,662,385 views, 318,749 likes, 1033 comments, and 54,245 shares from the included videos (Table 1). Physicians produced 57.4% of the included videos (*n* = 58), followed by patients (*n* = 24), nonphysician health care providers (physician assistants, registered nurses, advanced practice registered nurse) (*n* = 14), general content creators (*n* = 4), and other creators (*n* = 1) (Table 1). Ninety-two of the videos were educational, 8 were personal experience, and 1 was for advertising (Table 1). All personal experience videos were created by patients.

**Table 1.** Stats of Included Videos

	Number of videos	Total likes	Total comments	Median (IQR) Section 1 DISCERN score	Median (IQR) Section 2 DISCERN score	Median (IQR) Section 3 DISCERN score	Median (IQR) Total DISCERN score
Creator Type							
Physician	58	255,170	5936	28.0 (24.5-36.0)	19.0 (19.0-26.0)	3.0 (3.0-4.0)	58.0 (47.0-61.8)
Nonphysician care provider	14	12,623	1227	28.0 (25.0-36.0)	23.0 (20.8-26.0)	2.5 (2.0-3.5)	48.0 (38.8-57.5)
General content creator	4	9 93	124	21.5 (24.5-36.0)	32.0 (24.5-34.3)	4.0 (3.0-5.0)	64.5 (57.0-67.8)
Patient	24	19,487	2565	32.0 (24.5-36.0)	26.0 (19.0-32.0)	4.0 (3.0-5.0)	64 (44.8-66.0)
Other	1	30,476	481	25	23	4	52
Gender							
Male	50	246,438	5891	28.0 (21.3-28.9)	26.0 (18.3-32.0)	4.0 (3.0-5.0)	64.0 (42.3-66.0)
Female	51	72,311	4442	28.0 (25.0-36.0)	24.0 (19.0-32.0)	3.0 (3.0-5.0)	59.0 (50.0-65.0)
Other							
Video category							
Personal experience	25	19,873	2641	22.0 (18.9-28.0)	19.0 (12.0-27.0)	3.0 (2.0-4.0)	42.0 (32.0-59.0)
Educational	75	291,974	7614	28.0 (28.0-36.0)	26.0 (19.0-32.0)	4.0 (3.0-5.0)	64.0 (56.5-65.5)
Advertising	1	6902	78	25	21	3	49

When stratified by content creator type, median DISCERN scores were similar across groups, although nonphysician healthcare providers had the numerically highest median score, which was followed by physician, patient, and general content creator. There was no significant difference in the median total DISCERN score between the groups (*P* = .280, ε^2^ = 0.011). When assessing each of the components of the DISCERN score, there were noted to be significant differences for Item 7 (*P* = .040, ε^2^ = 0.030) with patients scoring higher than providers for providing additional sources of information, Item 9 (*P* = .004, ε^2^ = 0.050) with physicians and nonphysician care providers scoring higher than other groups in regards to describing how the treatment works, and finally Item 12 (*P* = .030, ε^2^ = 0.040) with physicians and nonphysician care providers scoring higher than other groups in regards to describing effects if no treatment was used. The groups scored overall low in the categories of areas of uncertainty and providing additional areas of support.

Male gender was associated with a higher median DISCERN score compared to female (64.0 vs 59.0), which was not statistically significant. When stratified by each category of the DISCERN scoring, there were no significant differences between the 2 groups. Finally, educational videos were noted to have a higher median DISCERN score, which was followed by personal experience and advertising (*P* > .05). When videos were stratified by video category type (personal experience, educational, etc.), there were noted to be significant differences in Item 7 (*P* = .007, ε^2^ = 0.094) with educational videos having fewer sources of support. In addition, educational resources were found to have significant differences in Item 9 (*P* = .005, ε^2^ = 0.107), item 10 (*P* = .049, ε^2^ = 0.052), and Item 12 (*P* = .049, ε^2^ = 0.053).

## DISCUSSION

The growing utilization of social media has led to social media platforms being used to disseminate and discuss health-related information. Of these platforms, TikTok has been shown to be a powerful resource that has gained traction in recent years.^14^ Our study is the first study to document the quality of health-related information on TikTok that is related to GLP-1 medications and plastic and reconstructive surgery. This study found a remarkable number of likes, comments, and shares in regard to plastic surgery and GLP-1-related comments, demonstrating the popularity of TikTok as a platform for the discussion of plastic surgery-related content.

Of the included videos in this analysis, physicians and other health-care providers posted the majority of videos (71.3%) followed by patients (23.8%). When videos were stratified by profession (physician, patient, etc.), we found that patients typically performed better than healthcare professions in question 7 (providing additional sources of support/information). This finding may be secondary to healthcare professionals feeling less of a need to include references from literature given the training that they have undergone in comparison to patients or companies who find the need to provide more support materials to help validate their opinions and information since they lack the commonly accepted certifying credentials that healthcare providers usually have and display. As a result, providers can improve the quality of their videos by including sources such as journal articles or other health information related videos for their videos to be more informative. Lastly, all groups were found to have scored low in the regions of uncertainty, which can be improved upon by providers discussing limitations regarding the unknown implications of undergoing GLP-1-based management for weight loss and the relation to plastic surgery, such as ozempic face.^15^

Educational videos received more likes, comments, and shares than advertisements or personal experiences, highlighting the potential for TikTok to aid in the dissemination of healthcare information. Also, educational videos scores higher in categories that were related to treatment description, benefits, and management with conservative therapy. Educational videos were primarily made by healthcare professionals in comparison to those made by patients, which was typically personal experience. However, other studies analyzing plastic surgery content on TikTok reported more robust engagement with nonphysicians and videos detailing personal experiences, perhaps due to a failure of physician-created content to reach target audiences or because consumers trust content from laypersons to the same degree as medically trained individuals.^16^ In either case, ensuring consumers and patients recognize the value of acting on trustworthy information provided by credible sources remains incredibly important.

Similarly, previous studies analyzing the quality of plastic surgery videos on TikTok have found that physician-created videos typically score higher than that of videos that are created by nonphysicians.^17,18^ In addition, it is imperative to recognize that regardless of the content creator type, low quality content has the ability to generate misinformation and influence individuals’ understanding of plastic surgery procedures and Ozempic. Given this, providers should be encouraged to inquire about patients’ preconceived impressions and their origins, including but not limited to social media content. Furthermore, providers should look to continue to create a welcoming and open environment with patients so that shared decision-making can occur along with transmission of healthcare information without any preconceived notions.

Our study is not without its limitations. At any given time, depending on an individual's prior search algorithm, the top 50 videos may change, making the videos included in our analysis different from those that another individual may identify. Although we took care to perform this study on a brand new TikTok account, there may still be subtle differences that can change the findings of this paper. Also, we selected a total of 4 search terms for our analysis, which were derived arbitrarily; as a result, it is possible that should additional, fewer, or different search terms have been used, the results of this study may be different, in regard to the content that was included and DISCERN scores for the respective groups. In addition, through our initial inclusion and exclusion criteria, the videos that were included in this analysis were inherently more informative and educational, as videos that did not meet the goal of discussing GLP-1-related medication and plastic surgery were excluded. As previously discussed, the excluded videos were analyzed, and there is the possibility that those videos may have been created by patients. Also, the majority of the videos included in this analysis were from healthcare providers rather than patients, which may once again skew some of our results.

## CONCLUSIONS

The impact of TikTok as a platform for medical education and patient outreach warrants further investigation into the quality and influence of videos relating to healthcare. Physician and nonphysician content creators should bear in mind the global reach of the platform, as well as the importance of providing accurate information. Methods to improve videos include providing qualified sources of healthcare information, fostering an environment of shared decision-making, and for providers to discuss how GLP-1 medications and plastic surgery may affect quality of life. Furthermore, to aim for both quality and popularity, creators should look to balance reliable content with an engaging format in order to garner viewership. While DISCERN may serve as a validated source of assessing health information, further research is needed to establish the tool's reliability in evaluating TikTok videos and possibly creating a scoring system that assesses specifically social media content.
